# Exploring critical care nurses' experiences of redeployment to general medical‐surgical wards for covering staff shortages: A qualitative research study

**DOI:** 10.1111/nicc.13267

**Published:** 2025-02-27

**Authors:** Naim Abdulmohdi, Yingchang Huang

**Affiliations:** ^1^ Faculty of Health, Medicine and Social Care, School of Nursing and Midwifery Anglia Ruskin University Cambridge UK; ^2^ Respiratory Support and Sleep Centre Royal Papworth Hospital Cambridge UK

**Keywords:** critical care nursing, patient safety, redeployment, staff experience, staff well‐being

## Abstract

**Background:**

The impact of redeployment on staff was not widely recognized before the COVID‐19 pandemic. The redeployment of critical care nurses is frequently employed in health care organizations as a last‐minute staffing management approach without proper infrastructure. Studies exploring the feelings and experiences of redeployed critical care nurses in this manner are sparse.

**Aim:**

The aim of this study was to develop an understanding of the experiences of critical care nurses during their redeployment to cover shifts in the general wards.

**Study Design:**

This study adopted a qualitative research design to explore critical care nurses' experiences of redeployment. Ten critical care nurses attending a postgraduate critical care course in one university were recruited between January and April 2019. Data were collected using a focus group which was thematically analysed.

**Results:**

Three themes were identified: ‘challenges in transitioning to ward settings’, ‘concerns regarding patient safety and satisfaction’ and ‘insufficient infrastructure and support’. Participants expressed concerns about patient and staff safety during redeployment, describing feelings of anxiety and undervaluation.

**Conclusions:**

The study illuminates the intricate challenges experienced by redeployed critical care nurses, highlighting the crucial need for comprehensive support, targeted training and strategic planning to safeguard patients’ care and nurses’ well‐being during periods of staff shortages in health care settings. These findings emphasize the importance of proactive measures in fostering a resilient and adaptable health care workforce. This is a small study involving only one focus group of 10 critical care nurses, which may limit the transferability of the findings. As a result, the findings may not fully reflect the experiences of critical care nurses from other sites.

**Relevance to Clinical Practice:**

The study reveals that critical care nurses experience challenges and stress when redeployed to general wards because of insufficient support and infrastructure. It emphasizes the need for comprehensive strategies, such as job rotation, tailored training and collaborative efforts to address redeployment challenges. Research could focus on developing and testing different models of job rotation between critical care and general wards, evaluating their impact on nurse satisfaction, confidence, competence and overall experience. The findings highlight the importance of organizational support, leadership and local guidelines for effective teamwork. Conducting longitudinal studies to track the experiences of redeployed critical care nurses over an extended period would be beneficial. This approach would provide insights into the long‐term effects of redeployment on job satisfaction, burnout and retention.

**What is known about the topic**

- Redeployment is a common practice in health care organizations during periods of crisis.
- The impact of redeployment on staff, particularly before the COVID‐19 pandemic, was not recognized, but studies during the pandemic have shown increased stress, anxiety and burnout among nursing staff.

**What this paper adds**

- This paper fills a gap in existing literature by specifically exploring the experiences of critical care nurses routinely redeployed to cover shifts in general medical‐surgical wards outside pandemic times.
- Redeployed staff found the experience very stressful, were concerned about personal and patient safety and satisfaction and reported insufficient support infrastructure.

## 1 INTRODUCTION

Redeployment is a common practice in health care organizations and was frequently employed during the COVID‐19 pandemic. However, the impact of redeployment on staff was not widely recognized before the pandemic, and there is a lack of clarity on the terminology. The primary objective of deployment is to ensure that the right personnel are in the right place at the right time to deliver high‐quality patient care. In contrast, redeployment is a strategy used to adapt to changing conditions or staffing challenges by reassigning health care workers to different roles or areas within the organization. Redeployment is considered a symptom of staff shortages, and while temporarily reassigning staff to another clinical area addresses these shortages, there is insufficient guidance on how to redeploy staff safely. Further development and evidence are necessary.

Redeployment during the COVID‐19 pandemic led to increased stress, anxiety and burnout among nursing staff, as indicated by various studies.^1^, ^2^, ^3^ Lasater et al.^4^ also found that nurses working in hospitals with lower nurse‐to‐patient ratios were more likely to report higher levels of burnout, an intention to leave their job and a lower quality of care. Multiple factors contributed to these negative outcomes, including changes in clinical instructions, concerns about patient and staff safety, inadequate organizational support, heavier workloads, lack of preparedness and recognition from leadership and insufficient training for redeployment.^1^, ^2^, ^5^ Although anxiety levels may have before and during the COVID‐19 pandemic and across different specialties and seniority levels, the common factors contributing to anxiety and the lack of support during redeployment are essential areas of investigation.^6^, ^7^ These emotions can also be triggered when nurses perceive a misalignment between care delivery and expected goals or encounter external factors that hinder optimal patient care, and when they sense a lack of control over the situation.^1^, ^8^, ^9^ Zimbudzi and Fraginal^10^ and Kennedy et al.^11^ identified common themes in staff experiences of redeployment, including anxiety, fear and concerns about unfamiliar clinical settings and the scope of practice. In Vera San Juan et al.'s^7^ study, staff experienced high levels of stress and anxiety from redeployment because of a lack of support or not having the opportunity to opt out of redeployment. However, these studies specifically focused on staff being redeployed to critical care during the pandemic, leaving a gap in evidence regarding the redeployment experiences of critical care nurses that are commonplace before and after the COVID‐19 pandemic.

Critical care units provide specialized care for patients with life‐threatening conditions. Managing these patients requires expertise, and relocating critical care nursing staff should be carefully considered and temporary to ensure patient safety across the organization. The shortage of skilled nurses poses a threat to patient safety and the delivery of high‐quality patient care.^12^ The redeployment of critical care nurses is frequently employed in health care organizations as a last‐minute staffing management approach without proper infrastructure. Although this redeployment is mostly temporary to cover one shift, it is usually frequent and intermittent. Studies exploring the feelings and experiences of redeployed critical care nurses in this manner are sparse. Furthermore, evidence confirms the detrimental impact of low staffing levels on omissions of essential nursing care and patients' outcomes (such as patients' mortality)^4^, ^13^, ^14^, ^15^, ^16^, ^17^; therefore, staff–patient ratios have been considered a key specification for critical care services in the United Kingdom.^18^ Nurses are professionally accountable for practising effectively, raising concerns and ensuring the safety of patients, the public and themselves. They must recognize when they are feeling overwhelmed, as this can potentially affect the quality of patient care.^19^ Kissel et al.^20^ investigated the impact of different learning models in supporting nurses redeployed to intensive care units (ICUs) and found that team‐based learning and support from critical care specialists were well‐received. However, in many cases, when critical care nurses are sent to the ward, training and teamwork are not implemented. Redeployment is usually left to managers with limited guidance and without recognition of the impact on the redeployed staff.

Before the COVID‐19 pandemic, research on nurse redeployment was scant, with only a few studies focused on critical care nurses being deployed in military services.^21^, ^22^ Matlakala^23^ is the only study on redeployment among critical care nurses conducted before COVID‐19. This study involved focus groups with critical care nurses and found that there was a lack of negotiation and consultation when assigning nurses to the ward, as they were redeployed without established procedures to guide the process. Karim et al.^5^ conducted a mixed‐methods study post‐COVID pandemic among nurses and health care workers, finding that redeployment was associated with both negative and positive feelings. Negative feelings included concerns about the risk to their professional registration, feelings of being unsafe, dissatisfaction and stress. O'Connor and Dugan,^24^ in their discussion paper on the use of floating, also highlighted similar issues related to staff satisfaction and stress during redeployment. Some of the positive feelings noted in Karim et al.'s study were the new learning opportunities gained through redeployment, but it also identified a lack of supportive organizational policies for redeployed staff and concerns about fairness in allocation.

To date, there has been little exploration into the experiences of critical care nurses regarding redeployment and deployment prior to the COVID‐19 era. Moreover, research on critical care nurses' encounters with redeployment during the COVID‐19 pandemic remains limited. Additionally, there is an assumption that critical care nurses are highly skilled in caring for various types of patients. Hence, it seems a safe decision to redeploy them to other units based on this assumption, regardless of the clinical specialty of the areas to which they are being redeployed. Furthermore, general wards differ in protocols, routines, resources and staffing compared to critical care settings, making it an unfamiliar environment for the redeployed staff. Previous research found that nurses' perceptions of their incompetence in working in an unfamiliar environment pose a risk to patient and staff safety and contribute to increased staff stress.^10^, ^11^, ^25^ During the second wave of the COVID‐19 pandemic, various practical recommendations were released, including those related to rapid cross‐skilling and evaluating safe staffing levels.^26^ However, not all of these recommendations were grounded in strong empirical evidence or staff experience.

A significant proportion of newly qualified nurses express their intention to leave the field or do so prematurely within 2 years of completing their education.^27^ Additionally, there is a high level of nursing vacancies, especially in UK health care services, where nurse vacancies make up more than a third of all job vacancies in this sector.^28^ These factors are likely to exacerbate the occurrence of redeployment and its negative impact on staff and their retention. Exploring the experiences of redeployed critical care staff is crucial for organizations to understand the underlying causes and implement appropriate policies and procedures to address these issues. It is worth noting that this research was conducted before the COVID‐19 pandemic, focusing on the experiences of critical care nurses redeployed to general wards.

## 2 AIM

The aim of this study was to develop an understanding of the experiences of critical care nurses during their redeployment to cover shifts in the general wards.

## 3 DESIGN AND METHODS

This study adopted a qualitative exploratory research design and followed an interpretivist approach to explore the experiences of critical care nurses through redeployment to cover shifts in general wards using a face‐to‐face focus group between January and April 2019. Thematic analysis was used to identify themes in the data. Standards for reporting qualitative research (SRQR) guidelines was used to report the study findings.^29^

### 3.1 Study settings and recruitment

All critical care nurses enrolled in a postgraduate course in 2019 at a large university in England were invited to participate. This group of participants was selected for this research because they shared a similar experience of being redeployed to a general ward to cover staff shortages. Additionally, having all the participants in one location provided an opportunity to invite and potentially recruit them, helping to mitigate the challenge of releasing critical care staff for research purposes. The second author (YH) invited the participants during their break and provided them with researcher's contact details. Interested participants contacted the second author, and the focus group meeting was conducted between teaching sessions in a pre‐booked room on theuniversity campus. The room was selected to ensure students' familiarity with the setting, maintain confidentiality and prevent distractions. In total, 10 critical care nurses from five different hospitals participated in the focus group. It was challenging to identify suitable times to organize two different focus groups, so the researchers made a pragmatic decision to conduct one focus group with 10 participants instead of two smaller groups.

### 3.2 Data collection

A face‐to‐face focus group interview was conducted, lasting approximately 60 min. The session was audio‐recorded with the participants' permission, and an interview schedule (Table 1) was used to encourage critical care nurses to describe their experiences during redeployment. Follow‐up questions were asked based on the topics and themes introduced by the interviewees.^30^ Participants were encouraged to speak freely and to respect each other's opinions during the focus group.

**TABLE 1:** Interview guide.

Could you please share your experience of working in the ward to cover staff shortages? Could you please share your feelings about working in the ward to cover staff shortages? How often have you been redeployed to the ward? How does your experience of redeployment to general wards affect your work in critical care? Could you please tell us about the support you received from leaders before and throughout the redeployment?

The second author YH, who conducted the focus group and took notes, was a senior staff nurse working in a critical care unit and a postgraduate student at the same university, with previous experience of being redeployed. Although the second author (YH) had worked with two participants in previous roles several years ago, they were in an advanced practice role outside the critical care setting and did not have direct clinical interaction with participants during this research. The first author (NA) worked as faculty in a school of nursing and was not directly involved in data collection, teaching or assessment of the participants. Both researchers have more than 10 years of experience in critical care, and the first author is an experienced researcher with postdoctoral research experience.

In our study, we recruited 10 participants into a single group instead of two as a pragmatic decision to maximize the number of participants. Despite the limitation of having a single focus group, the data provided a rich and in‐depth understanding of critical care nurses' experiences during redeployment. Guidance on data saturation in focus group studies is limited outside of the Grounded Theory methodology. Guest et al.^31^ found that most codes and themes emerged from the first focus group, indicating that thematic saturation occurs early. Similarly, Hennink, Kaiser and Weber^32^ agree that little additional benefit is gained beyond the second focus group.

### 3.3 Data analysis

The authors conducted thematic analysis following Braun and Clarke's^33^ approach. The audio and text files were examined, sometimes concurrently while taking notes, as a way to become familiar with the data. The transcripts were read in one session to develop data familiarity, and this process was repeated multiple times to immerse in and enhance analysis validity. The transcripts were imported into NVivo 12 software for data analysis. Transcribed data were individually and systematically coded line by line, resulting in a compilation of descriptive codes.^33^ These codes were subsequently organized to unveil recurring patterns. Sub‐themes were amalgamated to depict a reduced set of significant patterns representing the main themes. The same author (YH) conducted data collection, transcription and analysis, contributed to the credibility of the results. Both authors independently performed data analysis for confirmability, ensuring the requirements for findings to be corroborated by another researcher,^34^ and the team then discussed, reflected on and agreed on the final theme to further ensure the internal research validity and conclusions.

### 3.4 Trustworthiness

Study design and conduct were underpinned by Lincoln and Guba's^34^ ‘trustworthiness’ principles (credibility, transferability, dependability and confirmability of findings). The researchers kept a reflective diary throughout the data collection and analysis process. The researchers (NA and YH) frequently met during both data collection and analysis to discuss the decisions made and the content of the diary. A record of the methods was maintained to ensure the study's objectivity. Data analysis was conducted independently by the two researchers to facilitate cross‐checking. Both researchers are experienced in critical care nursing, with the first author being an experienced researcher. This enhances qualitative credibility and dependability.^35^

### 3.5 Ethical approval

Participation in this study was voluntary, and data were pseudonymized. All participants were provided with a participant information sheet (PIS) and asked to provide written informed consent before data collection began. The PIS outlined that participants could withdraw at any time without affecting their relationship with the university. Before the focus group proceedings began, participants were reminded of the study's purpose. The study received approval from the University Research Ethics Committee (approval number NMM‐SREP‐18‐003) on 7 January 2019, and institutional approval was granted by the course leader and senior managers at the univeristy.

## 4 FINDINGS

The study involved 10 critical care nurses, aged between 24 and 42. Their experience working in critical care units ranged from 2 to 8 years. All participants were staff nurses with similar job statuses. Table 2 shows that most of the participants were female, with five of them 30 years old or younger, and novice nurses with limited clinical experience were the most frequently redeployed staff, often for 12‐h shifts. The number of times critical care nurses were redeployed ranged from 3 to 10 per year. Most of the participants worked in three different general hospitals.

**TABLE 2:** Sample characteristics.

Characteristics		Number
Gender	Female	9
	Male	1
Age (years)	20–25	2
	26–30	3
	31–35	1
	36–40	2
	41–45	2
Years of experience in critical care	1–2	5
	3–4	3
	>4	2
Years of experience working in general wards before working in critical care	0–2	8
	3–4	2
Types of hospitals and critical care	District General	3
	General	6
	Specialist	1
Frequency of redeployment to general ward in the last year	0–5	4
	6–10	6

**FIGURE 1:**
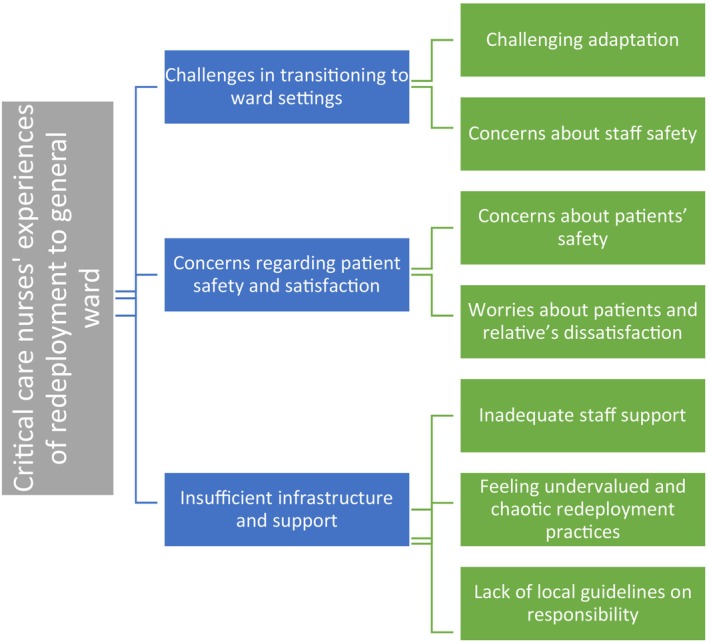
Themes.

The analysis revealed four major themes (see Figure 1). The content of the focus group interview delineated these themes, describing the experiences of critical care nurses when redeployed to general medical‐surgical wards.

### 4.1 Challenges in transitioning to ward settings

#### 4.1.1 Challenging adaptation

Most of the redeployed critical care nurses faced difficulties adapting to general ward settings. Many had not worked on a ward for years, leading to prolonged completion of tasks like drug rounds. Differences in nursing care, unfamiliar routines and new protocols hindered teamwork and patient care, causing frustration. The transition to the ward environment posed challenges similar to those faced by new nurses, yet without induction or supervision.

It's like a completely new job they should stop running places like that. If they do want that, maybe they should make us rotate and learn how to do it before we are moved. (Nurse 1)

#### 4.1.2 Concerns about staff safety

Most of the participants expressed concerns about their clinical responsibilities and accountability to patients, leading to heightened stress and negative feelings regarding personal safety. Some suggested regular rotation of critical care nurses to support the ward, emphasizing the need for ongoing training to ensure competence and confidence in these roles.

I have been a night shift in charge of the wards with an agency nurse. It was an area that I had never worked even as a student. Towards the end of the shift, I was so stressed; I just thought then and there, if it was like that, I am going to have to leave this job. (Nurse 3)

I am happy to support colleagues. However, at the same time, I also need to look after my PIN and myself; I am not going to help other people to be detrimental to myself. (Nurse 5)

### 4.2 Concerns regarding patient safety and satisfaction

#### 4.2.1 Concerns about patients' safety

Some participants highlighted that redeploying critical care nurses to general wards reduced safe staffing levels in critical care units. This resulted in instances where critically ill patients were not properly repositioned, potentially jeopardizing patient safety. Furthermore, the added responsibility of senior critical care nurses working at the patients' bedside, alongside their clinical and unit management duties, negatively impacted teamwork, staff support and care quality.

I have to go when the unit is very busy; we are left short of staff. […], the unit cannot function properly, they cannot get the patient moved and repositioned then you are taking away from the sickest patients in the hospital. (Nurse 2)

Participants expressed concerns and anxiety about patient safety during their redeployment from critical care to general wards. They mentioned instances of unsafe practices, such as unrealistic expectations from critical care nurses, including being asked to administer medications without proper handover. One redeployed nurse described witnessing a student nurse administering patient medication without adequate supervision from their mentor and expressed concerns about the potential detriment to the safety of both patients and the student nurse.

Some of the things I have seen require safeguarding. Such as there was a student nurse giving out medication without any supervision, she was on her own, I had to intervene and get the site manager to come, I asked her to hand in the keys to the medicine cabinet. (Nurse 5)

#### 4.2.2 Worries about patients and relatives' dissatisfaction

Many participants discussed their worries about patients' dissatisfaction and being perceived as incompetent by patients and their relatives during redeployment.

The relatives come to asked you about their mum needs and you are still reading the hand‐over to find out. You feel so incompetent and embarrassed […] you can't give the answer straight away. (Nurse 4)

### 4.3 Insufficient infrastructure and support

#### 4.3.1 Inadequate staff support

Most participants expressed empathy for the ward staff and a willingness to assist colleagues, but they felt let down by the support system. They pointed out a lack of leadership when relocating nurses within the hospital. Critical care nurses redeployed to the ward often worked with junior or agency staff, receiving limited support themselves. Although the matron in critical care was willing to send nurses to the ward, seeking support from site managers on the ward proved futile.

We do not have much support, and the staff there [in the wards] are so junior then there is even less support as other times which is really hard. (Nurse 3)

#### 4.3.2 Feeling undervalued and chaotic redeployment practices

It is also evident that the redeployed critical care staff felt undervalued and not listened to. Several participants discussed their dissatisfaction with the redeployment decision and felt that they had been unfairly treated.

Even when you put your hands up to say that you do not have any support and you are not comfortable with the situation. It feels there is no support and site manager come down and tell you that you do not have a choice but to get on with it. (Nurse 4)

It is more of the junior staff who get sent and sometimes, some of the junior staff gets sent all the time. It's always them who have to go. (Nurse 8)

Some participants discussed that the redeployment of staff was usually disorganized, with managers focused on filling numbers rather than carefully considering patient safety and staff competency. The process was often reactive, with staff sent to unfamiliar areas, sometimes with last‐minute changes. Some participants experienced multiple moves in a single shift to address staff shortages, and junior nurses were frequently relocated, potentially because of their more recent ward experience.

There is a lack the responsibility from the people above as a whole, they just randomly moving staff around. They do not know what is going on. It is like fighting a fire. I noticed we are not treated as nurses; it is more about numbers. (Nurse 8)

#### 4.3.3 Lack of local guidelines on responsibility

Some participants in the study emphasized the need for local guidelines to define the responsibilities of critical care nurses redeployed to the ward. Many critical care nurses lack recent ward experience, and the constant changes in ward protocols make immediate adaptation challenging for redeployed staff.

They need to have a set of guidelines to say what we can or cannot do. (Nurse 4)

They should have clear documentation to say certain things that you can or cannot do. Or you do these things if you are comfortable, do it by choice. (Nurse 9)

## 5 DISCUSSION

The purpose of this study was to explore the experiences of the redeployed critical care nurses to covering staff shortages in general medical‐surgical wards. The findings from the focus group identified three major themes: challenges in transitioning to ward settings, concerns regarding patient safety, and satisfaction and insufficient infrastructure and support.

In our study, participants expressed a sense of inadequacy when redeployed to general wards, feeling like novices. They faced challenges in swiftly adapting to the different working environment and the heightened expectations placed on them as critical care nurses. The unfamiliar settings in the general medical‐surgical wards, requiring distinct knowledge and skills, resulted in perceived incompetence and frustration among participants. This concurs with O'Connor and Dugan's^24^ on the challenges with floating and resonates with Kennedy et al.'s^11^ findings during the COVID‐19 pandemic, highlighting themes of nurses' specialization and challenges in unfamiliar settings. Similarly, Matlakala's^23^ study underscored a common misconception about nurses working in any setting without orientation, neglecting unique challenges. The disparities between ward and critical care nurses' roles, as highlighted by Häggström and Asplund,^36^ revealed challenges in cultural differences, varying competencies and communication uncertainties during transitions. Nurses' perceptions that their skills do not match the demands of their role or the needs of patients during redeployment could potentially have several negative consequences for the quality of care delivered, job satisfaction and turnover.^37^

Concurrent with our study, major stressors among redeployed critical care nurses were identified, including concerns about their safety, patient safety and satisfaction and the professional accountability of unsafe practices. These worries align with previous research,^1^, ^5^, ^6^, ^8^, ^9^, ^21^ indicating that misalignments between care delivery and expected goals, external factors hindering optimal patient care and a perceived lack of control contribute to such emotions. Our findings are consistent with Varcoe et al.,^25^ which reported that nurses felt distressed when working outside their scope of practice; Boyd,^21^ who highlighted a lack of pre‐deployment preparedness; and Abdulmohdi's^1^ research, which identified concerns about patient safety being associated with increased burnout levels. Zimbudzi and Fraginal^10^ identified anxiety, fear and concerns about unfamiliar clinical settings and scope of practice as common themes in staff experiences of redeployment, aligning with our study's findings. Karim et al.'s^5^ findings also resonate with our study, as they identified critical care staff's concerns about their safety, increased stress and anxiety and the impact of redeployment on the continuity of care. This is consistent with studies of redeployed staff during the COVID‐19 pandemic, which demonstrated a high level of anxiety during redeployment and an increased demand to effectively work in complex teams.^7^ Halter et al.^38^ reported that losing a sense of security, facing excessive workload and experiencing work‐related exhaustion have direct negative effects on turnover and job satisfaction. Nurse turnover reached high levels and intensified post‐COVID‐19 pandemic, exceeding 12.5%, with nursing vacancies accounting for 35% of all NHS vacancies.^39^ Improving working conditions, work engagement, pay and the perception of staff delivering good nursing care are important factors affecting nurse turnover.^40^

Participants shared experiences of distress, echoing Varcoe et al.'s^25^ findings, attributing this distress to structural failures and the added pressure of working with inexperienced or agency staff during redeployment, which potentially risks the safety of both patients and nurses. Participants in our study reported concerns that the practice of handing over critical care patients to the critical charge nurse conflicted with established guidelines, specifically the Guidelines for the Provision of Intensive Care Services.^18^ These guidelines mandate the presence of a supernumerary clinical coordinator and recommend additional registered nurses for units with more than 11 beds. Frequent redeployment of critical care nurses to the ward contributed to inadequate staffing levels in the ICUs and skills mismatch, potentially jeopardizing the care of critically ill patients. Participants in the current study described a sense of responsibility towards the critically ill patients in their base units and how their redeployment added more worries regarding their ICU patients' safety. They expressed sympathy for the ward's needs and also stressed the importance of a support system for safe and efficient patient care during redeployment for both wards and critical care patients.

The participants in this study reported insufficient infrastructure and support during their redeployment, impacting their intention to leave and the quality of patient care. This aligns with studies like Ballantyne and Achour's,^2^ which indicate that nurses feel abandoned, experience a lack of communication and receive inadequate support from managers. Abdulmohdi's study linked the perceived lack of organizational support to burnout^1^. Chang^41^ emphasized the importance of organizational support in nurses' job satisfaction. For mutual support during staff shortages, organizations must provide robust support for redeployed nurses. Participants also expressed dissatisfaction with managers, which aligns with Ballantyne and Achour's findings, highlighting the need for transparency in decision‐making. Leadership support, equitable allocation and guidelines are crucial, as confirmed by Burton et al.^42^ Nurse leaders play a positive role in problem‐solving and support, emphasizing the importance of consultation and skill assessment before redeployment. Cummings et al.^43^ also found that relational leadership, as opposed to task‐focused leadership styles, is linked to better nursing workforce outcomes and related organizational outcomes, such as nurses' job satisfaction, retention, individual productivity and a positive working environment. The Nursing and Midwifery Council^19^ also emphasizes the importance of ensuring that everyone to whom tasks are delegated is adequately supervised and supported to provide safe and compassionate care. There should not be an assumption that critical care nurses are capable of working in different clinical settings without adequate training and support.

Job rotation between general wards and critical care was suggested by some of the study participants. Job rotation broadens nurses' knowledge and skills, enhances workforce adaptability and boosts nurse confidence across various practice areas, positively impacting the working environment.^44^, ^45^ Alfuqaha et al.^46^ and Platis et al.^47^ identified positive correlations between job rotation, nurses' job satisfaction and job commitment, subsequently reducing turnover. Incorporating job rotation into a development programme tailored to specific clinical areas could serve as a solution to alleviate stress associated with redeployment.^48^, ^49^ Developing local guidelines for redeployment, as suggested by participants, is crucial for effective teamwork between ward and redeployed nurses. Valiee and Salehnejad^50^ emphasized the importance of encouragement, organizational support and training to enhance the development and adherence to clinical guidelines. Collaborative efforts between general wards and critical care nurses in developing local guidelines are essential for fostering mutual understanding and support during redeployment. Kissel et al.^20^ also found the importance of staff training and readiness before redeployment and likewise Abbott et al.^51^ suggested the use of a buddy system as an effective way to support redeployed staff. Best practices outlined by Critical Care Network National Nurse Leads^52^ also emphasize the need for orientation and supervision of critical care staff before redeployment to cover shifts in general medical‐surgical wards.

More attention is needed from nurse leaders and policymakers to enhance satisfaction and commitment in the work environment. Addressing insufficient infrastructure and support for redeployed nurses requires a multifaceted approach. This includes effective organizational support, consultation and skill assessment by nurse managers, collaborative development of local guidelines and exploration of job rotation benefits. Such a comprehensive strategy aims not only to safeguard job satisfaction but also to ensure the delivery of high‐quality patient care during critical care staff redeployment to the ward. The significant impact of redeployment on staff and retention further emphasizes the urgency of implementing evidence‐based strategies in health care settings.

### 5.1 Study strength and limitations

The limitations of the study include recruiting critical care nurses who were currently enrolled in a university course and conducting only one focus group, which may have impacted representation. Conducting two separate focus groups with five participants each, or considering individual interviews, might have enhanced the depth of the collected data. The study explored the experiences of critical care nurses redeployed to general medical‐surgical wards, contributing to the limited research available on this topic worldwide. Although we conducted only one focus group, it included 10 participants from five different hospitals, encompassing both secondary and tertiary settings. Participants had a diverse range of clinical experiences, enhancing the insights across these five different hospitals. The findings highlight the prevalent issue of staff shortages in the health care system and their impact on staff experiences. They may reflect similar challenges faced by hospitals redeploying critical care staff to address shortages nationwide. However, these results should be interpreted with caution, as the study was based on only one focus group. Both researchers are highly experienced critical care nurses familiar with redeployment but one of the researchers (YH) had previously worked in critical care with a few participants, which could introduce selection bias to participate. One researcher (YH) conducted the focus group and took notes. Although this did not impact the quality and flow of the discussion, having another researcher to take notes would have been useful to ensure nothing was missed.

### 5.2 Implications for practice and research

It will be helpful to conduct longitudinal studies to track the experiences of redeployed critical care nurses over an extended period. This approach would provide insights into the long‐term effects of redeployment on job satisfaction, burnout and retention. Further exploration of the concept of job rotation as a strategy to mitigate the challenges of redeployment is required. Research could focus on developing and testing different models of job rotation between critical care and general wards, evaluating their impact on nurse satisfaction, confidence, competence and overall experience.

## 6 CONCLUSION

In conclusion, this study explored the experiences of redeployed critical care nurses during staff shortages in general medical‐surgical wards. Three major themes emerged: challenges in transitioning to ward settings, concerns about patient safety and satisfaction, and insufficient infrastructure and support. Most of the participants expressed a sense of inadequacy and frustration, resonating with previous studies on nurses' specialization challenges. Major stressors included worries about safety, patient satisfaction and professional accountability. The conflict with established guidelines, inadequate staffing levels in ICUs and moral distress among participants highlighted structural failures and the need for orientation and supervision during redeployment. The study also emphasized the importance of organizational support, leadership and local guidelines for effective teamwork. The findings underscore the necessity for comprehensive strategies, including job rotation, to address the challenges of redeployment and enhance the satisfaction and safety of critical care staff. Effective communication and supportive leadership were deemed essential for positive team dynamics. The study urges proactive measures within the health care system to address staff concerns, enhance job satisfaction and mitigate further staff losses amid the current extreme staff shortage. This is a small study involving only one focus group of 10 critical care nurses, which may limit the transferability of the findings. As a result, the findings may not fully reflect the experiences of critical care nurses from other sites.

## Data Availability

The data that support the findings of this study are available from the corresponding author upon reasonable request.
